# Precautions with Regard to Puerperal Fever

**Published:** 1875-07

**Authors:** 


### MISCELLANEOUS.

### Precautions with regard to Puerperal Fever.

What, then, are the duties of the medical practitioner in whose practice a case of puerperal fever has occurred? He may change his clothes, he may take any number of baths, he may use all sorts of disinfectants, and still the fatal poison may hang about him, and he may be the bearer of it. Under these circumstances he should refrain from practicing midwifery for a season. It would be wantonly wicked in us were we to speak hesitatingly when such issues are at stake. The danger to the public arising from the non-observance of the precautions above named are incalculable, as we have seen in the outbreak in the neighborhood of the Wadsworth road and at Coventry, and whatever sacrifice the medical man may have to make in carrying out the precautions named, we unhesitatingly say he should make it. In the majority of cases, however, the sacrifice to be made would be light indeed. Three years ago an outbreak of puerperal fever took place in a provincial town of considerable size. On investigation it was found that all the cases had occurred in the practice of one surgeon and one midwife. The members of the profession located in the town met and requested the gentleman in question to refrain from practicing midwifery for one month, and at the same time agreed to attend his cases for him. The midwife was likewise requested not to attend cases for a similar period. This was agreed to, and no new cases occurred after that date. This illustrates well the line of conduct that should be adopted by the profession in any town where this terrible disease may happen to appear. The unfortunate practitioner in whose practice the case has occurred should bring it before the profession, and should cease from attending midwifery for a time. On the other hand, his professional brethren should help him through his difficulty, and make his loss as small as possible by attending his cases for him during the period of his abstention.—*London Lancet*, March, 1875.

